# The Impact of Environmental Degradation on Agricultural Crop Productivity: The Case of Pakistan With Simulated ARDL Approach

**DOI:** 10.1002/fsn3.70876

**Published:** 2025-09-03

**Authors:** Arshad Ullah Jadoon, Zixiang Zhao, Gizachew Wosene, Desalegn Wondim, Yan Yunxian

**Affiliations:** ^1^ School of Economics and Management Jilin Agricultural University Changchun City China; ^2^ School of Business and Management Jilin University Changchun Jilin Province China

**Keywords:** CO_2_ emissions, crop productivity, NDS‐ARDL, Pakistan

## Abstract

Environmental degradation is a cross‐cutting issue that significantly hampers the productivity of the agriculture sector in Pakistan. The objective of this study is to investigate the impact of environmental degradation on agricultural productivity in Pakistan from 1973 to 2021. The study employed the Novel Dynamic Simulated Autoregressive Distributed Lag (NDS‐ARDL) estimator as a data analysis technique to gauge the impact of environmental degradation (measured by CO_2_) and other variables, including agricultural credit, quality seeds, fertilizers, and the availability of irrigation water, on agricultural productivity. The major finding of this study shows that the impact of CO_2_ on crop productivity is negative but insignificant in both the short and long run. However, water availability, quality of seeds used, credit used for agriculture, and fertilizer use have a positive and significant impact in both the long and short run. Productivity improves with increased credit disbursement, quality seeds, and irrigation water. Based on these findings, policymakers should prioritize ensuring the stable and reliable availability of irrigation water supplies, promoting certified high‐quality seeds, expanding access to agricultural credits, discouraging ineffective land expansion, and planning for better land use to avoid the overuse of marginal lands, thereby enhancing crop productivity in Pakistan.

## Graphical abstract

The research, based on the NDS‐ARDL model, estimates the effect of environmental degradation on agricultural productivity of Pakistan over a period of 1973–2021. The results reveal that although CO_2_ emission has a negative impact on crop productivity, the application of fertilizers, high quality seeds and regular water supply significantly affect productivity in both short and long run. Access to agricultural credit and proper land use also have a profound impact on crop yields, emphasizing sustainable resource management as crucial to realizing agricultural returns.

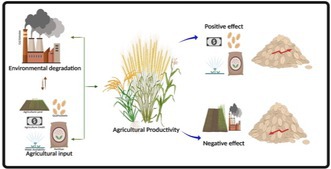

## 1 Introduction

The agricultural sector constitutes the largest component of the national economy for many developing countries (DCs) (Awan and Abro 2021; Dethier and Effenberger 2012; Siddiqui et al. 2012). It employs the highest proportion of DCs' labor force (Awan and Abro 2021) and fulfills the food needs of the economy (Hafeez et al. 2020). The sustainable rise in the economy's productivity over the long run depends on transforming the agricultural sector. It improves food security and enhances the quality of life of marginalized communities (Siddiqui et al. 2012).

The agricultural production function is directly influenced by climate change, among other inputs, including improved seeds, fertilizers, irrigation, and the availability of credit (Gupta et al. 2014). Climate change has been observed as a significant pressure on the productivity of the agricultural sector in recent decades in developing countries, especially in Pakistan. While previous studies have determined its negative impact on the productivity of the agricultural sector, a research gap remains in understanding the nonlinear and asymmetric effects of environmental degradation over a long period (Awan and Abro 2021; Tan et al. 2022). It contributes to the body of literature by employing a dynamic simulated ARDL model to examine the impact of both positive and negative shocks on productivity. It is for this reason that the global community is struggling to find ways to mitigate climate change and pollution levels without compromising agricultural productivity (Dethier and Effenberger 2012). It poses a serious threat to crop production and food insecurity (Abbas 2022; Abbas et al. 2022) and has devastating effects on health and the economy as a whole (Chandio et al. 2024). According to Tan et al. (2022), it is a vital challenge, among others, to feed the world's increasing population while maintaining the accelerated growth rate of the agricultural sector in the presence of environmental deterioration of the natural environment. Researchers are concerned about food security as a global challenge (Amin et al. 2014).

Although agricultural production is adversely affected by the deterioration of the natural environment, agriculture itself is a significant source of environmental pollution. In the study of Ashraf et al. (2024), they established the evidence that food production significantly contributes to pollution in Pakistan. Agriculture is one of the main sectors of the economy in Pakistan, with major crops including cotton, rice, sugarcane, wheat, vegetables, and fruits (Rehman et al. 2015). Growth in the agriculture sector determines overall economic growth, employment opportunities, and poverty levels in the country (Ministry of Finance 2023–2024). It enhances the GDP of the country by 23.5% and engages a total of 37% of the labor force (Ministry of Finance 2024–2025). However, limited empirical studies have been conducted on the impact of environmental degradation on agricultural productivity in Pakistan. Autoregressive Distributed Lag Model (ARDL) is used to evaluate the impact of climate change on the production of cotton and wheat in Pakistan from1980 to 2014 (Bibi and Naeem 2021). This study differs from the current study in time frame and data analysis techniques. Another group of researchers investigated the short‐run and long‐run impact of formal credit (CR) and climate change (CC via CO_2_ emissions) on agricultural production (AP) in Pakistan (Chandio et al. 2024). Other imperative control variables included in this study comprise technology factors (tractors [TRs] and tube wells [TWs], energy consumption [EC], and labor force [LF]) using the data covering the period 1983–2016. The autoregressive distributed lag (ARDL) approach was employed to examine the co‐integration between the underlying variables and used the Granger causality test under the vector error correction model (VECM) context to determine the direction of causality among the variables. These empirical studies differ from the current study in terms of the types of variables used, time frame, and data analysis techniques.

Others investigated the impact of variation in average annual air temperature and average annual precipitation on productivity using a two‐factor regression model. The main finding of the study reveals that temperature and precipitation have a negative impact on agricultural production (Ali et al. 2017). Chandio et al. (2024) used ARDL model to estimate the effect of climate change on the production of fruit in Pakistan, revealing that climatological factors such as temperature and CO_2_ emissions negatively affect apples output, while precipitation has a favorable impact on production in both the long and short run.

The empirical studies differ significantly from the current study in terms of the data's time frame, data analysis techniques, and the nature of the agricultural commodities. In this context, the present study contributes to the existing body of literature by examining the asymmetric influence of environmental degradation ($\mathrm{CO}2_{t}$) on the yields of crops (${\mathrm{CPI}}_{t}$) in Pakistan in the period 1973–2021. The same impact is assessed while controlling specification bias by including other explanatory variables such as agricultural land as a percentage of total land (${\mathrm{AL}}_{t}$), growth of credit disbursed to agriculture sector ($\text{Credit}\_G_{t}$), log of distribution of improved seeds in 000 tons ($\ln\_{\text{Seeds}}_{t}$), availability of water at millions of acres (${\mathrm{WAT}}_{t}$), and fertilizer consumption as a percentage of total fertilizer production (${\mathrm{FS}}_{t}$). The empirical analysis is undertaken using a Novel Dynamic Simulated Autoregressive distributed lag (DS‐ARDL) model proposed by Jordan and Philips (2018). This is a more useful model than the ARDL. It reports the asymmetric marginal effects of each explanatory variable, that is, positive and negative shocks, over the time horizon of short and long periods of time. Moreover, it can also automatically predict the decomposition, estimation, and simulation of shocks in the explanatory variables into negative and positive shocks. In other words, this is different from the prior studies by applying a non‐linear, dynamic simulated ARDL framework to quantify asymmetric effects of environmental degradation. In fact, prior studies have mainly focused on symmetric relationships, but the present methodology can capture real‐world variability more effectively. Moreover, it incorporates the most recent available data from 1973 to 2021, as well as underexplored variables such as seed quality and irrigation.

## 2 Review of Literature

The theory of production and environmental economics constitutes the theoretical foundation for analyzing the relationship between environmental degradation and the productivity of agriculture. The conventional production function of neoclassical economists postulates that land, labor, capital, and technology determine the output of the agriculture sector. However, environmental degradation, as measured by increased CO_2_ emissions along with these inputs, may either increase or decrease agricultural productivity, depending on threshold levels, farmer adoption strategies, and elasticities of substitution among the inputs. Moreover, a popular hypothesis, known as the Environmental Kuznets Curve (EKC), hypothesizes a U‐shaped relationship between environmental degradation and productivity, where initially, the environment degrades as productivity rises, and then declines after a certain productivity threshold is reached. This framework advocates for studying non‐linear and asymmetric relationships, as is done in this study.

The agriculture sector of Pakistan is subdivided into four sub‐sectors, which are crops, livestock, forestry, and fishing (Ministry of Finance 2023–2024). The crop sector is the major sector within the overall agricultural sector, which is dependent on environmental degradation, among other factors. It is believed that an increasing level of environmental degradation can negatively affect the agriculture sector's productivity. Some of the studies that support the above claim are Ali et al. (2017), Awan and Abro (2021), Chandio et al. (2024), Jan et al. (2021), Rehman et al. (2022).

Most studies have employed the ARDL model to analyze the marginal effects of explanatory variables on the dependent variable. Some of these studies are Abbas (2022), Abbas et al. (2021), Chaudhry et al. (2021), Ramzan et al. (2022), Shah et al. (2023). The studies are summarized below.

Chaudhry et al. (2021) examined the influence of climate change and environmental degradation on the crop production of Pakistan during 1977–2016. They analyzed the data using the ARDL model. They concluded that the growth rate of average temperature has a significant direct effect on the yields of crops in shorter and longer periods. In contrast, the growth rate of CO_2_ emissions has a significant direct impact in the long run, but its effect is found to be negative and insignificant in the short run. However, Awan and Abro (2021) concluded that the climate index has a negative impact on wheat and sugarcane yields from the panel data of four provinces of Pakistan from 1993 to 2019. In Pakistan, the CO_2_ emissions adversely affect the productivity of the agricultural sector from 1961 to 2018 using the ARDL model (Ramzan et al. 2022).

The impact of climate change on fruit yields in Pakistan has been investigated from 1991 to 2020. The analysis is conducted within the framework of the ARDL model. The major results confirmed that CO_2_ emissions reduce apple yields, whereas a positive impact is established to produce bananas, mangoes, guavas, and mangosteens (Chandio et al. 2024).

Climate change: rising temperature has adversely affected crop production in the period of 2000–2019 in Pakistan. The analysis indicates that the coefficient is statistically significant in the longer period but not in the shorter period. Fertilizers and seed quality have demonstrated a positive impact on crop production (Abbas 2022). In another study, Arooj et al. (2018), similar findings were also reported. Siddiqui et al. (2012) found a non‐negative coefficient of climate change for wheat production. These findings are based on district‐level panel data from the province of Punjab for 1980–2008.

There has been an indirect relationship between average temperature, an indicator of climate change, and crop productivity, as reported by Gul et al. (2022) in Pakistan for the period 1985–2016. They also revealed that the fertilizer and credit variables promote the overall productivity of the country. Similar results are also reported by Ali et al. (2017), Baig and Amjad (2014), Jan et al. (2021), Rehman et al. (2022) for Pakistan. They used annual time series data. Moreover, Baig and Amjad (2014) forecast extreme events and weather for the period 2010–2020 using the vector autoregressive (VAR) model. Abbas et al. (2022) examined the influence of the average minimum and average maximum temperatures on various regions of Punjab, Pakistan, from 1979 to 2018 using the non‐linear ARDL model. They confirmed that the influence of climate change on rice yield is asymmetric. They also reported that the average maximum over the long run had negative and inverse impacts on rice yields. Similarly, the earlier study of Amin et al. (2014) reported that the average minimum and maximum temperatures adversely and significantly affect the production of various types of rice in Bangladesh.

Similar to the study by Bhardwaj et al. (2022), Jan et al. (2021) also investigated the impact of average minimum and maximum temperatures on selected crops in the Indian Punjab, they found that the minimum temperature has a positive effect on the yield of wheat and rice, whereas the maximum temperature has an inverse influence on the crops. Chandio et al. (2022) examined the negative impact of CO_2_ emissions in the short run on the yields of various crops in India from 1965 to 2015. In the other study, Baig et al. (2022) reported that the yield of rice has been inversely affected by mean temperature over the longer period. They studied the case of India for the period from 1991 to 2018. In the NARDL framework, they report the asymmetric effect of climate change on rice productivity, similar to Abbas et al. (2022).

It was revealed that the influence of climate change on the level of rice and maize output in Pakistan from 1993 to 2015 is negative and significant (Hafeez et al. 2020). Similarly, others investigated the influence of climate change on food and non‐food crops in India from 1961 to 2017 (Guntukula 2020). They established that food and non‐food crops, except rice, are adversely affected by average maximum temperature. They also report that the average minimum temperature positively affected food crops, whereas its impact is negative for non‐food crops. In the earlier study of Birthal et al. (2014), they established the favorable effect of a rise in minimum temperature on the yields of food crops of India in the time period of 1969 and 2005. They also report that an increase in maximum temperature has an adverse effect on food crop productivity. Gupta et al. (2014) investigated the influence of temperature, fertilizers, and irrigation water on the yield of food crops in India during 1966 and 1999. They conclude that rice yields are adversely affected by average temperature, whereas the average temperature square has a positive impact on the same variable. They also establish that fertilizer and irrigation promote rice yields in the country. In the case of the production of pearl millet crops, the impact of average temperature is found to be positive, and the effect of the average temperature square is established to be negative. They also found that fertilizer negatively affects the production of pearl millet.

Increasing temperatures can negatively affect food and non‐food crop production. It has been reported by Praveen and Sharma (2020) studied 15 types of leading crops over an extended period of 50 years, that is, between 1967 and 2016. Shah et al. (2023) explored how the agriculture sector responded to CO_2_ emissions and air pollution. They collected panel data from five BRICS countries for the period from 1990 to 2019. These include Brazil, Russia, India, China, and South Africa. They conclude that the productivity of the agricultural sector is negatively affected by CO_2_ emissions and air pollution. Amaefule et al. (2023) examined the Nigerian case of effects of climate change via the channel of CO_2_ emission on the value added by the agriculture sector for the period of 1960–2019. They analyzed the data using the second‐generation Environmental Kuznets Curve. They concluded that CO_2_ emissions and CO_2_ intensity negatively affect agricultural productivity, thereby creating a physical risk to the agricultural sector.

Using panel data from 35 European countries, Tan et al. (2022) explored the impact of loss of biodiversity, deforestation, and agricultural sector emissions on the productivity of the agricultural sector. They concluded that vegetable production is positively affected by agricultural sector emissions, whereas cereal production is negatively affected.

In summary, this study used the NDS‐ARDL model to estimate the impact of environmental degradation on crop productivity in the presence of control variables. Most studies use linear regressions, ARDL models, or panel data regressions. It is in this context that the present study is the first attempt to fill the above research gap. Therefore, it is a useful contribution to the literature in predicting the impact of explanatory variables in response to positive and negative shocks over a time horizon of 30 years.

## 3 Materials and Methods

The present study was conducted to examine the influence of environmental degradation on the productivity of the agricultural sector in Pakistan using simulated ARDL. The value added to major crops in Pakistan during the past 49 years ranged from 1973 to 2021. The functional relationship is shown in Equation (1), and the description of the variables, along with sources of data, is given in Table 1.

(1) $${\mathrm{CPI}}_{t}=f\left({\mathrm{FS}}_{t},{\mathrm{AL}}_{t},\mathrm{CO}2_{t},\text{Credit}\_G_{t},\ln\_{\text{Seeds}}_{t},\ln\_{\text{Water}}_{t}\right)$$

**TABLE 1:** List of variables with data sources.

Variable	Symbol	Definition	Source
Productivity of major crops	${\mathrm{CPI}}_{t}$	It measures crop production	WDI
Environmental degradation	$\mathrm{CO}2_{t}$	It is the CO_2_ emissions from gaseous fuel consumption (% of total CO_2_ emission)	WDI
Fertilizer consumption	${\mathrm{FS}}_{t}$	It the consumption of fertilizer (percentage of fertilizer production)	WDI
Agricultural land	${\mathrm{AL}}_{t}$	Agricultural land (% of land area)	WDI
Credit disbursement	$\text{Credit}\_G_{t}$	Growth of total credit disbursed to agriculture sector. It is reported in percentage	Various issues of economic surveys of Pakistan
Quality of seeds	$\ln\_{\text{Seeds}}_{t}$	It is measured by the natural log of distribution of improved seeds to agriculture sector. It is measured in 000 tons	Various issues of economic surveys of Pakistan
Status of water availability	$\ln\_{\text{Water}}_{t}$	It is natural log of total amount of water availability to agriculture sector measured as Million Acre Feet (MAF)	Various issues of economic surveys of Pakistan

*Note:* The analysis period spans from 1973 to 2021.

CPI is the dependent variable indicating the major crops (wheat, rice, and maize) cultivated in Pakistan. The selection of control variables, such as ${\mathrm{FS}}_{t}$, ${\mathrm{AL}}_{t}$, $\text{Credit}\_G_{t}$, $\ln\_{\text{Seeds}}_{t}$ and $\ln\_{\text{Water}}_{t}$ and is based on the relevant literature. In the literature, these are highlighted as vital explanatory variables for explaining crop yields. For instance, the studies of Cen et al. (2020), Guo et al. (2022), Zhai et al. (2022) have highlighted that fertilizer is one of the key variables mediating the infertility issue of land. They highlighted the use of various types of fertilizers to increase crop yield. On the other hand, Beillouin et al. (2020), Waha et al. (2020), Warsame et al. (2021) used the area under cultivation and assessed its effect on the crop yields. In similar lines, the studies of Bahşi and Çetin (2020), Balana and Oyeyemi (2022), D'souza (2020), Rasheed et al. (2024) motivated the use of disbursement of credit to the agriculture sector. Finally, the quality of seeds and irrigation water can enhance crop yields, as discussed by Dietz et al. (2021) and Liliane and Charles (2020).

In time series analysis, it is vital to know the unit root processes of the series, as it can determine the suitability of the technique for analysis. In the present case, Dickey and Fuller (1979) and Phillips and Perron (1988) tests were used to determine the order of integration of the time series. These tests are expressed by Equations (2) and (3). In Equations (2) and (3), and are constant terms, and t indicates the trend component in these equations. The null hypothesis is =1 and = 1 under the ADF and PP tests, respectively, meaning that the order of integration of the series is one, that is, I (1). Finally, and are random innovations of the series. Under the ADF, is assumed to be homoscedastic and serially uncorrelated. However, in the PP Test, is assumed to be I (0) and heteroscedastic.

(2) $$∆y_{t}=\alpha_{0}+\alpha_{2}t+\alpha_{1}y_{t-1}+\sum_{i=1}^{p}\beta_{i}∆y_{t-1}+\varepsilon_{t}$$

(3) $$∆y_{t}=\pi_{0}+\pi_{1}t+\pi_{2}y_{t-1}+\epsilon_{t}$$

The functional relationship expressed in Equation (1) is shown in Equation (4), following Pesaran et al. (2001) model of ARDL. It is a very popular model in economic analysis; however, Philips (2018) showed that when the number of periods is less than 80, the ARDL Bound test is very conservative. Moreover, the distinction between I (0) and I (1) is mandatory. In the prerequisites of implementation of the ARDL model, as Philips (2018) highlights, the condition of I (1) for the dependent variable is that the order of integration of explanatory variables is not higher, that is, I (2) or more, and the existence of no autocorrelation in the innovations of the model.

In the context of Philips (2018) critique, Jordan and Philips (2018) introduced a new way of estimating the ARDL model, given in Equation (4), known as the Novel Dynamic Simulated ARDL model.

(4) $$\begin{array}{c} ∆{\mathrm{CPI}}_{t}=\alpha_{0}+\theta_{0}{\mathrm{CPI}}_{t-1}+\theta_{1}{\mathrm{FS}}_{t-1}+\theta_{2}{\mathrm{AL}}_{t-1}+\theta_{3}\mathrm{CO}2_{t-1}\\ \;+\theta_{4}\text{Credit}\_G_{t-1}+\theta_{5}\ln\_{\text{Seeds}}_{t-1}+\theta_{6}\ln\_{\text{Water}}_{t-1}\\ \;+\sum_{i=1}^{P}\alpha_{i}∆{\mathrm{CPI}}_{t-1}+\sum_{j=1}^{q_{1}}\beta_{1j}∆{\mathrm{FS}}_{t-j}+\sum_{j=1}^{q_{2}}\beta_{2j}∆{\mathrm{AL}}_{t-j}\\ \;+\sum_{j=1}^{q_{3}}\beta_{3j}∆\mathrm{CO}2_{t-j}+\sum_{j=1}^{q_{4}}\beta_{4j}∆\text{Credit}\_G_{t-j}\\ \;+\sum_{j=1}^{q_{5}}\beta_{5j}∆\ln\_{\text{Seeds}}_{t-j}+\sum_{j=1}^{q_{6}}\beta_{6j}∆\ln\_{\text{Water}}_{t-j}+\mu_{t} \end{array}$$

In Equation (4), the null hypothesis ($H_{0}$) that no long‐run relationship exists between ${\mathrm{CPI}}_{t}\;\text{and}\;\left({\mathrm{FS}}_{t},{\mathrm{AL}}_{t},\mathrm{CO}2_{t},\text{Credit}\_G_{t},\ln\_{\text{Seeds}}_{t},\ln\_{\text{Water}}_{t}\right)$ is given by $H_{0}:\theta_{0}=\theta_{1}=\theta_{2}=\theta_{3}=\theta_{4}=\theta_{5}=\theta_{6}=0$. The Bound Test is distributed as *F*‐ or Wald—statistics under the null hypothesis. However, the bound test under a one‐sided *t* distribution can be used to test co‐integration such that $H_{0}:\theta_{0}=0$ (no co‐integration) under the alternative hypothesis ($H_{1}$) of $H_{1}:\theta_{0}<0$. The critical values for the *F*‐ and *t*‐distributions are available in Pesaran et al. (2001). The critical values of *F*‐ and *t*‐distributions depend on the number of regressors, at level forms, and the number of restrictions on constant and trend terms. Jordan and Philips (2018) highlight that the ARDL model in Equation (4) may have a fairly complex lag structure of dependent and independent variables. These variables may appear in lag, level, or first difference form. They introduced a new estimation method to dynamically simulate several autoregressive distributed lag (ARDL) models. It can estimate, simulate, and automatically plot predictions from ARDL models. They used stochastic simulation techniques to display the responsiveness of the dependent variable to the counterfactual change in one independent variable at a single point in time if all else were assumed to be unchanged. Jordan and Philips (2018) further identified that the use of stochastic simulation techniques requires that the order of integration and best fit of the ARDL model is determined using an appropriate information criterion or theory. This is necessary because the resulting residuals are assumed to be white noise and free from autocorrelation. In the first step, they estimated (4) using the ordinary least squares technique. In the second step, 1000 simulations or more are taken from a multivariate normal distribution for the vector of parameters. The null hypothesis that $H_{0}:\theta_{0}=\theta_{1}=\theta_{2}=\theta_{3}=\theta_{4}=\theta_{5}=\theta_{6}=0$ can be tested for each simulation. The assumptions regarding the mean and variance of these distributions are that the estimated parameters of the regressions are identical to the means, and the estimated variance–covariance matrix is equal to the variance. They further simulated variance (${\hat{\sigma }}^{2*}$) to bring back stochastic uncertainty into the model for the creation of predicted values. Draws from the scale inverse chi‐square distribution ($\chi^{2}$) were used to estimate ${\hat{\sigma }}^{2*}$. To ensure that the draws of ${\hat{\sigma }}^{2*}$ are bounded by 0 and 1, they scaled $\chi^{2}$ by the residual of $\left(n-k\right)$ degrees of freedom and the estimated ${\hat{\sigma }}^{2}$ from the regression. Finally, they used the values of the simulated parameters and ${\hat{\sigma }}^{2}$ to create the values for the dependent variable over time for each simulation by setting the values of the other explanatory variables equal to their respective means.

The neoclassical production function, such as *Y* = *f* (*K*, *L*) A is not estimated in the present study because of constraints related to data for Pakistan. Crop‐specific capital, labor, and technology data are not available in the country. It is for this reason that widely available and policy‐relevant proxies, such as fertilizer use, agricultural land, credit, water, and seed quality, have been used in the analysis. The dynamic simulated ARDL model enables simulations of the dynamic effect of explanatory variables on crop productivity, even with data limitations.

While agriculture both contributes to and is affected by CO_2_ emissions, the present study is not designed to capture bi‐directional causality. The focus is on how exogenous shocks in environmental degradation affect productivity. Future studies could incorporate structural VAR or simultaneous equation models to assess endogeneity.

## 4 Results and Discussion

**FIGURE 1:**
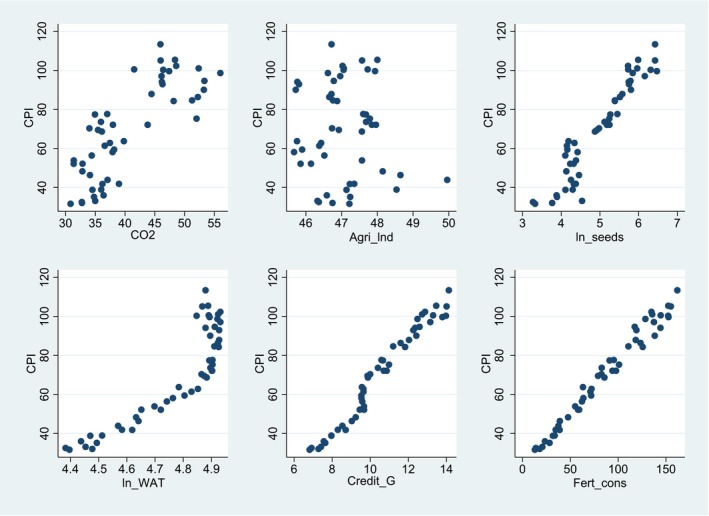
Scatter plot graph. *Source:* Author's calculation using EView13.0.

The descriptive statistics of all variables are given in Table 2. There is a total of 49 years of time‐series data. The descriptive statistics are self‐explanatory and supported by the scatter plots shown in Figure 1. It can be observed that $\ln\_{\mathrm{WAT}}_{t}$, $\text{Credit}\_G_{t}$ and $\ln\_{\text{Seeds}}_{t}$ show a direct and positive relationship with ${\mathrm{CPI}}_{t}$ respectively. However, there is no clear relationship between ${\mathrm{FS}}_{t}$, ${\mathrm{AL}}_{t}$ and $\mathrm{CO}2_{t}$ and ${\mathrm{CPI}}_{t}$.

**TABLE 2:** Summary statistics.

Variables	Observation	Mean	SD	Minimum	Maximum
${\mathrm{CPI}}_{t}$	48	69.7163	24.4817	31.6300	113.4200
${\mathrm{FS}}_{t}$	48	86.1104	44.3605	12.9022	161.9302
${\mathrm{AL}}_{t}$	48	47.0458	0.8766	45.6698	49.9546
$\mathrm{CO}2_{t}$	48	40.8820	7.1979	30.8321	56.0146
$\text{CREDIT}\_G_{t}$	48	17.5514	16.5316	−13.0343	68.9674
$\mathrm{Ln}\_{\text{SEEDS}}_{t}$	48	4.9633	0.8602	3.2699	6.47177
$\mathrm{Ln}\_{\mathrm{WAT}}_{t}$	48	4.7716	0.17403	4.38277	4.93152

*Source:* Author's calculation using Eview13.0.

Table 3 summarizes the correlation matrix among the variables. It is evident that all the explanatory variables show positive correlation with the dependent variable, ${\mathrm{CPI}}_{t}$ except ${\mathrm{AL}}_{t}$. It is also evident that $\mathrm{CO}2_{t}$ shows weak positive correlation with, ${\mathrm{CPI}}_{t}$. On the other hand, the negative correlation of ${\mathrm{AL}}_{t}$ with ${\mathrm{CPI}}_{t}$ is also reported. Theoretically it is believed that more area under cultivation shall results in increasing crop production as per increasing return to scale. However, it is also believed that the agricultural sector exhibits decreasing returns to scale in most cases, especially in developing countries, where agricultural practices are still outdated and not keeping pace with the speed observed in other developed countries. Other correlations coefficients can be explained in the same manners.

**TABLE 3:** Results of correlation matrix.

Variables	${\mathrm{CPI}}_{t}$	$\mathrm{CO}2_{t}$	${\mathrm{AL}}_{t}$	${\mathrm{FS}}_{t}$	$\text{Credit}\_G_{t}$	$\ln\_{\text{Seeds}}_{t}$	${\mathrm{WAT}}_{t}$
${\mathrm{CPI}}_{t}$	1						
$\mathrm{CO}2_{t}$	0.0166	1					
${\mathrm{AL}}_{t}$	−0.1250	−0.1462	1				
${\mathrm{FS}}_{t}$	0.4969	0.1094	−0.3314	1			
$\text{Credit}\_G_{t}$	0.1411	−0.2292	0.0760	0.1244	1		
$\ln\_{\text{Seeds}}_{t}$	0.2316	0.3151	0.0218	0.1188	0.3656	1	
$\ln\_{\mathrm{WAT}}_{t}$	0.0149	−0.0479	0.1052	−0.3593	−0.1299	0.0104	1

*Source:* Author's calculation using EView13.0.

The first prerequisite for estimating the dynamic simulated ARDL model was to test the unit root processes in the data. The order of integration was determined using ADF and PPerron tests (Table 4). It is shown that all these variables have unit roots at level, except ${\mathrm{AL}}_{t}$ as per the results of the ADF test. Similarly, the PPerron test indicates that all variables are stationary at level, except ${\mathrm{AL}}_{t},$ ${\mathrm{WAT}}_{t}$ and $\text{Credit}\_G_{t}$. This shows that all these variables are a mix of I (0) and I (1), which is a prerequisite for the application of dynamic simulated ARDL. Moreover, the order of integration of the dependent variable ${\mathrm{CPI}}_{t}$, is one, that is, I (1).

**TABLE 4:** Results of ADF and PPerron test of unit roots.

Variable	ADF test	PPerron test	I
${\mathrm{CPI}}_{t}$	−6.943 (0.000)	−16.876 (0.000)	I (1)
${\mathrm{FS}}_{t}$	7.356 (0.000)	−15.362 (0.000)	I (1)
${\mathrm{AL}}_{t}$	−5.342 (0.000)	−4.272 (0.001)	I (0)
$\mathrm{CO}2_{t}$	−5.704 (0.000)	−5.616 (0.000)	I (1)
$\text{Credit}\_G_{t}$	−6.344 (0.000)	−6.352 (0.000)	I (0)
$\mathrm{Ln}\_{\text{Seeds}}_{t}$	−11.206 (0.000)	−12.237 (0.000)	I (1)
$\mathrm{Ln}\_{\text{Water}}_{t}$	−5.867 (0.000)	−7.447 (0.000)	I (I)

*Note:* Probabilities are in parenthesis. I (1) means integrated of order one and I (0) means integrated of order zero.

*Source:* Author's calculation using EView13.0.

The numbers of lags for the ADF and PPerron tests were zero and three, respectively. In the next step, it is vital to select an appropriate lag length for the ARDL model. Several lag‐length selection criteria can be used. Table 5 lists the estimated outputs for some of these criteria. This study used the SBIC criterion, which was also suggested by Philips (2018) and Jordan and Philips (2018). The SBIC suggests that a lag length of one is appropriate for estimating the ARDL model.

**TABLE 5:** Lag order selection criteria.

Lag	LogL	LR	FPE	AIC	SC	HQ
0	−662.755	NA	19966.25	29.766	30.047	29.871
1	−445.490	357.278*	11.589*	22.288*	24.536*	23.126*
2	−406.909	51.441	21.579	22.751	26.967	24.323
3	−353.296	54.804	27.696	22.546	28.729	24.851

*Source:* Author's calculation using Eview13.0.

*indicate optimal lag order selection.

The coefficients of the ARDL model are presented in Table 6. This shows that the coefficient of ${\mathrm{CPI}}_{t-1}$ is positive and statistically different from zero at the 5% level of probability. This means that the current level of production is directly affected by the production in the previous year. In other words, the study reports that production levels in current and previous years are statistically interrelated.

**TABLE 6:** Results of ARDL (4, 2, 3, 0, 0, 2, 1) model.

Variable	Coefficient	SE	*t*‐Statistic	*p*
${\mathrm{CPI}}_{t-1}$	0.4252332	0.1644326	2.5860632	0.0156629
$\mathrm{CO}2_{t}$	−0.2614437	0.1790558	−1.4601237	0.1562349
${\mathrm{AL}}_{t}$	0.0463787	0.6914562	0.0670740	0.9470359
${\mathrm{FS}}_{t}$	0.3116056	0.0831854	3.7459153	0.0009037
$\text{Credit}\_G_{t}$	0.0212927	0.0322615	0.6600060	0.0150547
$\mathrm{Ln}\_{\text{Water}}_{t}$	69.084032	23.747635	2.9090910	0.0073303
$\mathrm{Ln}\_{\text{Seeds}}_{t}$	7.6820319	2.7221455	2.8220503	0.0090245
C	15.354089	45.403801	0.3381674	0.7379523

*Note:* *N* = 48. $R^{2}$ = 0.9923. Adj. $R^{2}=$ 0.9870. Log likelihood = −94.22443.

*Source:* Author's calculation using Eview13.0.

The fertilizer's use is found to be statistically significant and positively effecting crop productivity. It means that the use of fertilizers can significantly increase the level of crop production in Pakistan. The coefficient of ${\mathrm{FS}}_{t}$ is 0.3116056 (Table 6). This shows that a one‐unit increase in fertilizer consumption can increase crop production by 0.3116056 units. These results are as per the studies of Abbas (2022), Gupta et al. (2014) and Rehman et al. (2019).

The use of agricultural land as a percentage of the total land (${\mathrm{AL}}_{t}$) can be another control variable for variations in crop production. An increase in the ${\mathrm{AL}}_{t}$ every year can also increase the level of production of various crops. However, there may be diminishing returns to scale, as this is a very common phenomenon in the agriculture sector. The size of its coefficient is 0.0463787 and its sign is positive (Table 6). This means that a one‐unit increase in agricultural land leads to an increase in production by 0.0463787 units. These results are consistent with theories and study of Ejeta and Bai (2025) reported in the agriculture sector and statistically significant at conventional level of probability.

Environmental degradation measured by gaseous fuel consumption (percentage of total), $\mathrm{CO}2_{t}$ is the main variable of interest in the present case. The objective of this case study was to determine whether increasing $\mathrm{CO}2_{t}$ reduces crop production. The magnitude of the coefficient is −0.2614437 (see Table 6). This shows that a one‐unit increase in the level of $\mathrm{CO}2_{t}$ decreases the level of crop production by −0.2614437 units and its coefficient is not statistically significant. These results is consistent with the finding by Chandio et al. (2022).

Credit disbursed to the agriculture sector ($\text{Credit}\_G_{t}$) is key to influencing the level of crop production. As a matter of fact, most of the farmers in Pakistan are poor. They lack sufficient funds to purchase high‐quality seeds and fertilizers. They also lack the resources to improve and maintain the quality of agricultural land. The coefficient of $\text{Credit}\_G_{t}$ is 0.0212927 (see Table 6). This suggests that the disbursement of credit to the agricultural sector is highly beneficial in enhancing crop production levels. Its coefficient is statistically significant at the 5% level of significance. The studies of Iqbal et al. (2003), Rasheed et al. (2024), Rehman et al. (2017), and Rehman et al. (2019) also reported similar results.

The production of various crops depends on the availability of high‐quality seeds (Table 6). In this study, the coefficient of $\ln\_{\text{Seeds}}_{t}$ is 7.6820319. This indicates that the distribution of high‐quality seeds leads to an increase in the level of crop production in the country. These empirical findings are statistically significant at the conventional probability level of 1%. Liliane and Charles (2020) and Rehman et al. (2019) also reported that the distribution of better‐quality seeds can enhance productivity in the agricultural sector.

In the agricultural sector, the availability of a sufficient amount of water ($\ln\_{\text{Water}}_{t}$) play a vital role in increasing the country's production level. The coefficient of $\ln\_{\text{Water}}_{t}$ is 69.084032. This is statistically different from zero at the 1% level of significance. This indicates that consistent and regular flow of water for irrigation purposes promotes crop productivity. These findings are similar to those reported by Gupta et al. (2014) and Jan et al. (2021).

The ARDL model in Table 6 is used to decompose the marginal effects of ${\mathrm{FS}}_{t},{\mathrm{AL}}_{t},\mathrm{CO}2_{t},\text{Credit}\_G_{t},\ln\_{\text{Seeds}}_{t}\;\text{and}\;\ln\_{\text{Water}}_{t}$ into coefficients for shorter and longer periods of time. The estimated coefficients are presented in Table 7. The coefficients of $\mathrm{CO}2_{t}$ and ${\mathrm{AL}}_{t}$ are negative in the long term. This reveals that these variables reduce the crops' value‐added over a longer period in the country. It may be because of diminishing return to scale and land degradation. As matter of fact, most of the newly land brought under cultivations are low fertile and non‐irrigated land which can significantly reduce productivity. The coefficients of ${\mathrm{FS}}_{t}$, $\text{Credit}\_G_{t}$, $\ln\_{\text{Seeds}}_{t}$ and $\ln\_{\text{Water}}_{t}$ are positive, indicating the expansionary effects of these variables on crop productivity. However, all the coefficients are statistically different from zero, except for the coefficient of $\text{Credit}\_G_{t}$ which is insignificant.

**TABLE 7:** Error correction ARDL (4, 2, 3, 0, 0, 2, 1).

Variable	Coefficient	SE	*T*	*p* > |*t*|
(a) *Long run coefficients*				
${\mathrm{CPI}}_{t-1}$	−0.8999	0.1919	−4.687	0.0001
$\mathrm{CO}2_{t}$	−0.0149	0.1168	−0.1277	0.8993
${\mathrm{AL}}_{t}$	−2.4029	0.8071	−2.9768	0.0050
${\mathrm{FS}}_{t}$	0.3116	0.0831	3.7459	0.0009
$\text{Credit}\_G_{t}$	0.0212	0.0322	0.6600	0.5151
$\mathrm{Ln}\_{\text{Seeds}}_{t}$	7.3997	2.9028	2.5491	0.0170
$\mathrm{Ln}\_{\mathrm{WAT}}_{t}$	20.640	7.0103	2.9443	0.0015
Constant	17.0601	51.0494	0.3341	0.7409
(b) *Short run coefficients*				
$∆{\mathrm{AL}}_{t}$	1.1796	0.6262	1.8836	0.0684
$∆\mathrm{CO}2_{t}$	−0.2614	0.1385	−1.8865	0.0680
$∆\text{Credit}\_G_{t}$	0.0236	0.0372	0.6345	0.5351
$∆\mathrm{Ln}\_{\text{Seeds}}_{t}$	−0.2822	1.7227	−0.1638	0.8708
$∆\mathrm{Ln}\_{\mathrm{WAT}}_{t}$	69.0840	16.5652	4.1704	0.0002
CointEq	−0.8999	0.0966	−9.3136	0.0000

*Note:* (a) *N* = 48. $R^{2}$  = 0.7244. Adj. $R^{2}$ = 0.5337. *F*‐statistic = 3.7983. Prob (*F*‐statistic) = 0.0010. (b) *N* = 45.$R^{2}$ = 0.7244. Adj. $R^{2}=$0.6326. *F*‐statistic = 7.8888. Prob (*F*‐statistic) = 0.000002.

The short‐run effects of the explanatory variables on crop production are also given (see Table 7). This shows that effect of $∆\mathrm{Ln}\_{\text{Seeds}}_{t}$ have a negative effect on crop productivity in the short run, and not statistically significant. It indicates that they do not support the agricultural sector in the short term. On the other hand, the coefficients of $∆{\mathrm{AL}}_{t}$, Credit_G and $∆\mathrm{Ln}\_{\text{Water}}_{t}$ are positive and statistically significant. It means that effects of ${\mathrm{AL}}_{t}$ on crop production varies across the various lag structure of the variables. In other words, enhancing the area under cultivations exhibits expansionary effects at lag one and two but not at the current values. On the other hand, and $∆\mathrm{Ln}\_{\text{Water}}_{t}$ influence crop productivity positively in the short run.

It means that availability of regular supply of water plays crucial role in enhancing the overall crop productivity. The studies have reported that irrigation systems can significantly enhance crop productivity in Pakistan. Some of these papers are those in line with our present study such as (Gupta et al. 2014; Jan et al. 2021). Those studies distinguished the impact of fertilizer consumption on crop productivity over shorter and longer periods. Table 7 indicates that ${\mathrm{FS}}_{t}$ promotes productivity in the long run.

It is of high concern for countries to meet the higher demand for food and non‐food agricultural products. One of the variables among others is the discovery and distribution of high‐yield seeds to accelerate productivity. In this study, $\ln\_{\text{Seeds}}_{t}$ was incorporated as an explanatory variable because of its vital role in agriculture. It has been reported that $\ln\_{\text{Seeds}}_{t}$ increases the output in the long run which is also in accordance with the study of (Singh et al. 2013).

Table 8 presents the results of the cointegration tests. There are two tests of co‐integration *F*‐ and *t*‐statistics with the critical values corresponding to stationary critical values, that is, I (0), and non‐stationary critical values, that is, I (1), which were reported by (Pesaran et al. 2001). There is no cointegration among the variables if the value of the *F*‐statistic is lower than I (0). However, cointegration exists if the value of the *F*‐statistic is higher than I (1). In Table 8, it can be concluded that there is no evidence of co‐integration as per the *F*‐statistic and the *t*‐test.

**TABLE 8:** Co‐integration test.

10%		5%		1%	
I (0)	I (1)	I (0)	I (1)	I (0)	I (1)
2.218	3.314	2.618	3.863	3.505	5.121
2.188	3.254	2.591	3.766	3.540	4.931
1.990	2.940	2.270	3.280	2.880	3.990

*Note:* *F*‐statistic = 8.542894. No level relationship, that is, no co‐integration. If *F* < critical values for the I (0) regressors, accept and reject if *F* > critical values for the I (1) regressors.

*Source:* Author's calculations using Eview13.0.

In the final step, it is vital to test the model using diagnostic tests. Popular and conventional tests are estimated. To ensure the robustness of findings, multicollinearity diagnostics indicate that there is no multicollinearity, as evident from the correlation matrix in Table 3. The other tests indicate that the model does not suffer from autocorrelation, heteroscedasticity, or misspecification in the regression equation (see Table 9).

**TABLE 9:** Diagnostic tests.

Test	Coefficient	Probability	Decision
Breusch Godfrey LM test for autocorrelation	$\chi^{2}$ = 0.253	0.4697	No serial correlation
Breusch–Pagan–Godfrey test for heteroscedasticity	$\chi^{2}$ = 0.999	0.3155	No evidence of heteroscedasticity
Jarque–Bera statistic	2.731073	0.2552	Normally distributed since *p* > 0.05

The empirical results presented in Tables 4, 5, 6, 7, 8, 9 suggest that the novel dynamic simulated model can be applied in the present setting, as all the variables are either I (0) or I (1). The analysis further indicates that none of the variables are of a higher order of integration, and a feasible ARDL model is specified based on SBIC. Moreover, the same ARDL model indicates evidence of long‐run equilibrium relationships among the variables with clean diagnostic tests. Table 10 presents the empirical results for the same model.

**TABLE 10:** Dynamic ARDL model.

CPI	Coefficient	SE	*t*	*p* > *t*
${\mathrm{CPI}}_{t-1}$	0.1041718	0.1650129	0.63	0.532
$∆\mathrm{CO}2_{t}$	−0.1336739	0.2189523	−0.61	0.546
$\mathrm{CO}2_{t-1}$	−0.0153781	0.1431211	−0.11	0.915
$∆{\mathrm{AL}}_{t}$	−0.4774307	0.7439656	−0.64	0.525
${\mathrm{AL}}_{t-1}$	−1.716908	0.9125179	−1.88	0.069
$∆{\mathrm{FS}}_{t}$	0.255391	0.0867551	2.94	0.006
${\mathrm{FS}}_{t-1}$	0.2550839	0.0917411	2.78	0.009
$∆\text{Credit}\_G_{t}$	0.0238705	0.0402211	0.59	0.557
$\text{Credit}\_G_{t-1}$	0.0150291	0.0613024	0.25	0.808
$∆{\mathrm{WAT}}_{t}$	47.50746	26.14065	1.82	0.078
${\mathrm{WAT}}_{t-1}$	21.69366	7.388829	2.94	0.006
$∆\ln\_{\text{Seeds}}_{t}$	4.116618	2.987681	1.38	0.178
$\ln\_{\text{Seeds}}_{t-1}$	9.411529	3.480026	2.70	0.011
_cons	−27.73513	52.82253	−0.53	0.603

The empirical findings indicate that the two‐year lag impact of crop production on current production is positive and statistically insignificant. The coefficient is 0.10417. This means that bumper crop production in the current year will be followed by increased production in the coming 2 years. This is a common phenomenon in the agricultural sector, as highlighted in the literature.

The effect of the area of agricultural land ${\mathrm{AL}}_{t}$ is shown in Table 10. The coefficient of $∆{\mathrm{AL}}_{t}$ and ${\mathrm{AL}}_{t-1}$ indicates that an increase in agricultural land decreases the crop production. The coefficients of both ${\mathrm{AL}}_{t-1}$ and $∆{\mathrm{AL}}_{t}$ are negative. The impact of can be better evaluated if differences in soil quality, fertility, and availability of irrigation are present. In the overall mix, if the proportion of non‐irrigated, less fertile, and untreated cultivable land increases, it may reduce the aggregate crop yield in the country. It follows from the above discussion that considering such dimensions can help understand its influence on crop yield in the country.

The effects of environmental degradation are presented in Table 10. The coefficients of CO_2_ indicate that crops' production is negatively affected by the whether it is defined by $∆\mathrm{CO}2_{t}$ or $\mathrm{CO}2_{t-1}$. However, these coefficients were not statistically insignificant. These results are as per the literature.

On the other hand, the influence of fertilizer consumption on crop production is positive at $∆{\mathrm{FS}}_{t}$ and ${\mathrm{FS}}_{t-1}$ levels but statistically significant. It is as per theory that fertilizer consumption can have a positive effect on agricultural productivity in both short and long run.

It is widely believed that farmers in Pakistan are poor and cannot afford to buy high‐quality seeds, fertilizers, pesticides, and so on. They were unable to prepare their land for cultivation. In this context, it has been suggested that the provision of easy credit to farmers can significantly increase the sector's output. This view is supported by the coefficients of $∆\text{Credit}\_G_{t},\text{Credit}\_G_{t-1},∆\ln\_{\text{Seeds}}_{t}$ and ${\text{Seeds}}_{t-1}$. It can be observed that all the coefficients of the above variables are positive, indicating that an increase in credit provision to the agricultural sector can lead to increased crop production. However, among these coefficients, only ${\text{Seeds}}_{t-1}$ is statistically significant when the level of significance is set at 5%.

Table 10 also shows the impact of the availability of water for irrigation purposes on the value added by the crops in Pakistan. The variables $∆{\text{Water}}_{t}$ and ${\text{Water}}_{t-1}$ represent the availability of irrigation water. These coefficients indicate that the regular flow of water for irrigation purposes increases the level of production of crops in the country. These coefficients are statistically significant. The coefficients $\text{Credit}\_G_{t}$, $\ln\_{\text{Seeds}}_{t}$ and ${\text{Water}}_{t}$ are consistent with those reported in the literature.

**FIGURE 2:**
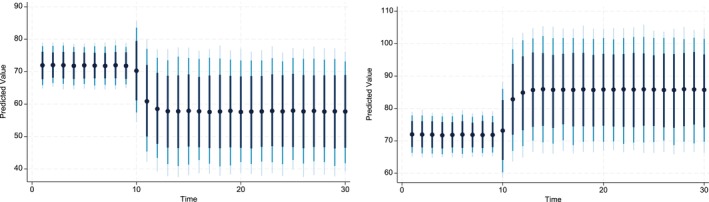
Dynamic simulated graph of ARDL for ${\mathrm{AL}}_{t}$.

**FIGURE 3:**
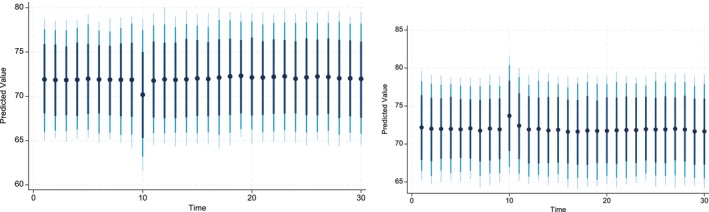
Dynamic simulated graph of ARDL for $\mathrm{CO}2_{t}$.

**FIGURE 4:**
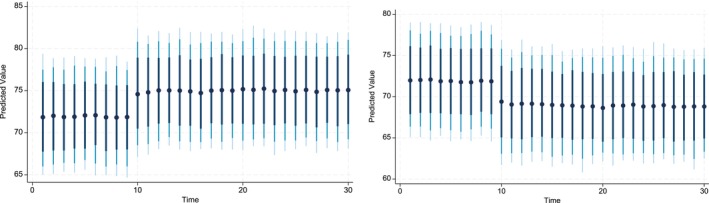
Dynamic simulated graph of ARDL for ${\mathrm{FS}}_{t}$.

**FIGURE 5:**
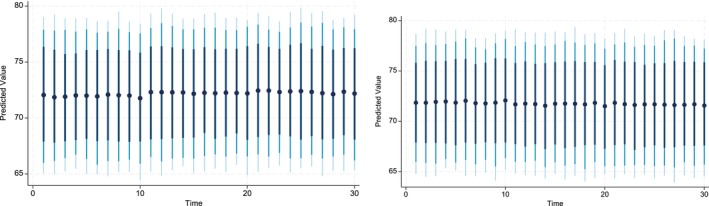
Dynamic simulated graph of ARDL for ${\text{Credit}}_{t}$.

**FIGURE 6:**
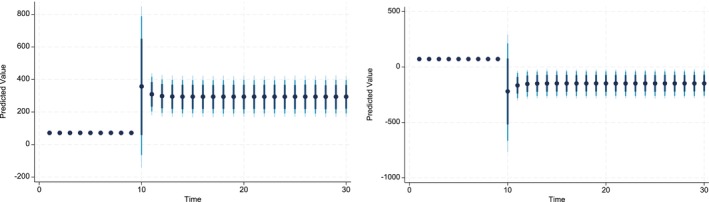
Dynamic simulated graph of ARDL for ${\text{Water}}_{t}$.

**FIGURE 7:**
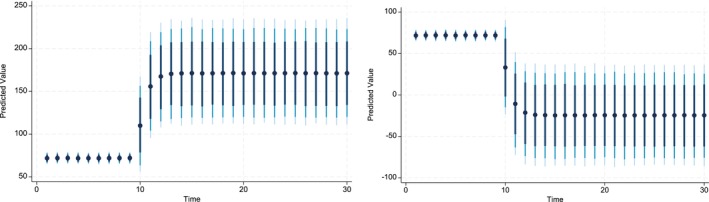
Dynamic simulated graph of ARDL for ${\text{seeds}}_{t}$.

The key advantage of using Dynamic Simulated ARDL over the simple ARDL model is that it simulates shocks in the explanatory variable. The present study simulated the shocks in each explanatory variable by a 10% increase or decrease over a 30‐year time horizon. The dots in each graph indicate the predicted value by the average obtained from the dynamic simulated ARDL model, whereas the shaded line shows confidence intervals of 75%, 90%, and 95%, respectively. These graphs are labeled in Figures 2, 3, 4, 5, 6, 7.

It has been established that high‐quality seeds can significantly improve overall production in the agricultural sector. In the present case, a variable representing better seeds is introduced in the model for the above reason. The empirical findings in Table 10 highlight that the influence of $∆\ln\_{\text{Seeds}}_{t}$ and $\ln\_{\text{Seeds}}_{t-1}$ on the value added by the crops in Pakistan is positive.

The 10% negative and positive shocks to the variable ${\mathrm{AL}}_{t}$ are shown in Figure 2. It can be observed that the predicted impact of shocks to ${\mathrm{AL}}_{t}$ on ${\mathrm{CP}}_{t}$ is negative in the shorter and longer periods. The predicted values are statistically insignificant. Moreover, a 10% negative shock to ${\mathrm{AL}}_{t}$ indicates that its impact on ${\mathrm{CP}}_{t}$ is constant up to the first 9 years, gradually increases up to 13 years, and then remains constant. On the other hand, a 10% positive shock reveals that its impact on ${\mathrm{CP}}_{t}$ remains unchanged up to the first 9 years. However, in the 10th year, the impact diminishes until the 13th year and finally becomes constant over the remaining prediction horizon until the 30th year.

The present study also predicted the responses of ${\mathrm{CPI}}_{t}$ to shocks in the $\mathrm{CO}2_{t}$ variable. These shocks can be negative or positive. The responses to ${\mathrm{CPI}}_{t}$ can differ from these shocks over a longer time horizon. The Figure 3 indicates that the long‐run and short‐run influence of a 10% positive or negative shock at the level of $\mathrm{CO}2_{t}$ has a direct and insignificant influence on the level of ${\mathrm{CPI}}_{t}$ in Pakistan. These predicted values were very similar in terms of their magnitudes.

Figure 5 shows the impact of negative and positive shocks to the $\text{Credit}\_G_{t}$ over the time horizon of 30 years. The predicted values indicate that 10% percent negative shocks to $\text{Credit}\_G_{t}$ produce a positive impact on the ${\mathrm{CPI}}_{t}$ it is statistically significant. However, after the time horizon of nine, the shocks produce a negative impact on ${\mathrm{CPI}}_{t}$ and are statistically significant. On the contrary, the 10% positive shocks to $\text{Credit}\_G_{t}$ have a positive impact on the ${\mathrm{CPI}}_{t}$ and, it is statistically significant in the short run and highly statistically significant in the long run. In the case of ${\mathrm{FS}}_{t}$, the dynamic simulated graph reveals the direct and significant predicted response of ${\mathrm{CPI}}_{t}$ to ${\mathrm{FS}}_{t}$ in both the short and long run (see Figure 4). In addition, the magnitude of the predicted response is similar, irrespective of whether the shock is positive or negative.

**FIGURE 8:**
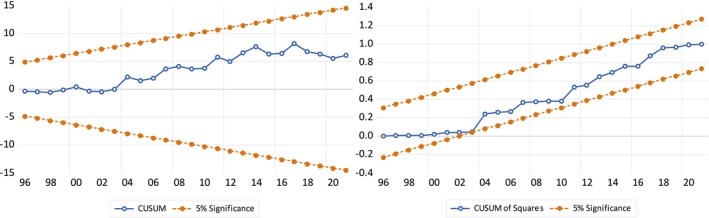
Stability test (CUSUM and CUSUM of square).

The dynamic impact of ${\mathrm{WAT}}_{t}$ on ${\mathrm{CPI}}_{t}$ is also positive whether shocks are negative or positive over the time horizon of 30 years (see Figure 6). The impulse response graph of the predicted values indicates that the response of ${\mathrm{CPI}}_{t}$ diminishes, after the 10th year, to the negative shocks in ${\mathrm{WAT}}_{t}$. Finally, the simulated response of $\ln\_{\text{Seeds}}_{t}$ to negative and positive shocks indicates that the predicted coefficients are positive in both the long run and short run (see Figure 7). These predicted values are statistically significant, except for the short‐run predicted values of a 10% increase in the $\ln\_{\text{Seeds}}_{t}$. Moreover, 10% negative shocks show that positive predicted values decrease as time approaches 30, whereas they increase slightly up to the time horizon of 13 years and then become stationary until the 30‐year horizon.

In the last step, CUSUM and CUSUM of Square tests were used to check whether these results were stable. The interpretation of these tests was straightforward. If the blue line is within a confidence interval of 5%, it is concluded that the model is stable. In the present case, Figure 8 shows that the model was stable. The Ramsey Reset test shows no specification error in the model (see Table 8).

The overall empirical evidence suggests that investing in high‐quality seeds and efficient irrigation systems substantially improves gains in productivity. However, bringing more area under cultivation without significantly updating technological know‐how or fertility tests of soil quality may lead to diminishing returns. Policymakers should prioritize the quality of resources over the quantity of resources.

## 5 Conclusions and Recommendations

The present study was conducted to assess the impact of environmental degradation on the productivity of major crops in Pakistan from 1973 to 2021, that is, 49 years of analysis. The analysis was conducted in the framework of the Novel Dynamic Simulated ARDL (DS‐ARDL) model developed by Jordan and Philips (2018). It is the most recent and new variant of the ARDL model that can test long‐run co‐integration relationships; distinguish marginal effects into short‐ and long‐run; and dynamically estimate, simulate, and predict the impact of negative and positive shocks to explanatory variables on crop productivity over the selected future time horizon.

The productivity of major crops was measured by the index of crop production. There are several measures of environmental degradation, but the present study used CO_2_ emissions from gaseous fuel consumption as a percentage of the total land. Several control variables are required to avoid model misspecifications. These control variables were the consumption of fertilizer, agricultural land as a percentage of total land, growth of disbursement of credit to the agriculture sector, quality of seeds, and availability of irrigation water.

The present study is the first attempt to apply the DS‐ARDL model to estimate, simulate, and automatically plot the predictive response of crop productivity to negative and positive shocks of magnitude 10% to all explanatory variables. This study used stochastic simulation techniques of DS‐ARDL developed by Jordan and Philips (2018) for a time horizon of 30 years with 1000 simulations.

The empirical results of this study show that the short‐ and long‐run impacts of CO_2_ emissions on crop productivity are negative. The short‐term effect of CO_2_ emissions on crop productivity is statistically insignificant in the ARDL model. On the other hand, NDS‐ARDL simulations indicate that positive and negative shocks to CO_2_ emissions, by a magnitude of 10%, predict a negative and insignificant impact on crop productivity in Pakistan over the next 30 years of predictions and simulations. However, the effect of fertilizer use on crop productivity is positive and significant. It indicates that the use of fertilizer has expansionary effects on crop productivity. The study also estimated the impact of the area under cultivation on the productivity of major crops. In the present case, its impact is negative. Its coefficient is negative and insignificant; in the case of DS‐ARDL, it is significant at ARDL. Moreover, the disbursement of credit, the quality of seeds, and water availability positively affect the productivity of major crops in Pakistan.

The graphs of DS‐ARDL show that productivity gains from areas under cultivation are significantly stagnant up to the prediction of 10 years, irrespective of the positive or negative shock. However, DS‐ARDL indicates that the predicted productivity of major crops rises as there is a negative shock to the area under cultivation, and vice versa. Regarding the role of credit issued to the agriculture sector, the simulated graph of DS‐ARDL indicates that its predicted impact is insignificant up to the initial 10 years of prediction. Moreover, a negative shock to agriculture's credit predicts a significant decline in expected productivity up to the 14th year, after which it becomes stagnant. However, the positive shock has a significantly favorable impact on crop productivity between 10 and 14 years and becomes stagnant thereafter.

The simulations show that the use of fertilizer positively, uniformly, and significantly affects the predicted crop productivity in the country over the next 30 years. Its overall impact on the DS‐ARDL model was positive and statistically significant. Finally, the simulated graphs of the availability of water for irrigation and quality of seeds indicate that a positive shock is accompanied by an increase in productivity and vice versa in the case of negative shocks. The predicted values were statistically significant.

To improve agricultural productivity in Pakistan while addressing environmental degradation, policymakers should design and adopt a feasible strategy that ensures sustainable resource management and supports farmers' access to crucial inputs.

The first thing the government should ensure is the stable availability of irrigation water through renovated water conservation techniques, such as drip irrigation and rainwater harvesting, to address water scarcity.

Secondly, the government can better work to improve the use of high‐quality, climate‐resilient seed varieties through research, subsidies, and on‐farm training programs, which can significantly improve cereal crop productivity.

Thirdly, the government should provide access to agricultural credit with limited bureaucracy, particularly for smallholder farmers, which will enable investment in modern farming technologies and equipment, ensuring long‐term productivity growth. Furthermore, the application of appropriate fertilizers should be used to prevent soil degradation and environmental harm. By implementing these measures, Pakistan can improve its agricultural productivity while mitigating the adverse effects of environmental degradation.

The compelling evidence of the asymmetric effects of environmental degradation on crop productivity is not without limitations. Some of the limitations include the exclusion of capital, labor, and technology as inputs from the model due to the unavailability of crop‐specific data, and the inability of nonlinear dynamic simulated ARDL to capture feedback loops between agriculture and CO_2_ emissions. Future research may explore ways to accommodate inputs of labor, capital, and technology. It may also include panel data approaches for more consistent estimates of the coefficients, along with structural equation modeling to resolve endogeneity issues and feedback effects of explanatory variables and crop productivity. Nevertheless, the results offer clear guidance: prioritize water availability, seed innovation, and credit access over land expansion.

## Author Contributions

**Arshad Ullah Jadoon:** conceptualization (lead), data curation (lead), formal analysis (lead), funding acquisition (lead), investigation (lead), methodology (lead), resources (lead), software (lead), validation (lead), visualization (lead), writing – original draft (lead), writing – review and editing (lead). **Zixiang Zhao:** conceptualization (equal), data curation (supporting), formal analysis (supporting), funding acquisition (equal), investigation (supporting), methodology (supporting), resources (equal), software (lead), validation (supporting), visualization (lead), writing – original draft (supporting). **Gizachew Wosene:** conceptualization (supporting), data curation (supporting), formal analysis (supporting), investigation (supporting), methodology (supporting), resources (supporting), software (supporting), supervision (supporting), validation (supporting), writing – original draft (supporting), writing – review and editing (supporting). **Desalegn Wondim:** data curation (supporting), formal analysis (supporting), investigation (supporting), methodology (supporting), validation (supporting), visualization (supporting), writing – review and editing (supporting). **Yan Yunxian:** conceptualization (equal), data curation (equal), formal analysis (equal), funding acquisition (lead), investigation (lead), methodology (lead), resources (equal), software (lead), supervision (lead), validation (equal), visualization (lead), writing – original draft (equal), writing – review and editing (equal).

## Conflicts of Interest

The authors declare no conflicts of interest.

## Data Availability

The data supporting the findings of this study are available from the corresponding author upon request, subject to reasonable conditions.
